# Involvement of the adaptor protein 3 complex in lignocellulase secretion in *Neurospora crassa* revealed by comparative genomic screening

**DOI:** 10.1186/s13068-015-0302-3

**Published:** 2015-08-20

**Authors:** Xue Pei, Feiyu Fan, Liangcai Lin, Yong Chen, Wenliang Sun, Shihong Zhang, Chaoguang Tian

**Affiliations:** College of Plant Sciences, Jilin University, Changchun, 130062 China; Key Laboratory of Systems Microbial Biotechnology, Tianjin Institute of Industrial Biotechnology, Chinese Academy of Sciences, Tianjin, 300308 China

**Keywords:** *Trichoderma reesei*, RUTC30, *Neurospora crassa*, Adaptor protein 3 complex, Lignocellulase secretion

## Abstract

**Background:**

Lignocellulase hypersecretion has been achieved in industrial fungal workhorses such as *Trichoderma reesei*, but the underlying mechanism associated with this process is not well understood. Although previous comparative genomic studies have revealed that the mutagenic *T. reesei* strain RUT-C30 harbors hundreds of mutations compared with its parental strain QM6a, how these mutations actually contribute to the hypersecretion phenotype remains to be elucidated.

**Results:**

In this study, we systematically screened gene knockout (KO) mutants in the cellulolytic fungus *Neurospora crassa*, which contains orthologs of potentially defective *T. reesei* RUT-C30 mutated genes. Of the 86 deletion mutants screened in *N. crassa*, 12 exhibited lignocellulase production more than 25% higher than in the wild-type (WT) strain and 4 showed nearly 25% lower secretion. We observed that the deletion of *Ncap3m* (NCU03998), which encodes the μ subunit of the adaptor protein 3 (AP-3) complex in *N. crassa*, led to the most significant increase in lignocellulase secretion under both Avicel and xylan culture conditions. Moreover, strains lacking the β subunit of the AP-3 complex, encoded by *Ncap3b* (NCU06569), had a similar phenotype to Δ*Ncap3m*, suggesting that the AP-3 complex is involved in lignocellulase secretion in *N. crassa*. We also found that the transcriptional abundance of major lignocellulase genes in Δ*Ncap3m* was maintained at a relatively higher level during the late stage of fermentation compared with the WT, which might add to the hypersecretion phenotype. Finally, we found that importation of the *T. reesei ap3m* ortholog *Trap3m* into Δ*Ncap3m* can genetically restore secretion of lignocellulases to normal levels, which suggests that the effect of the AP-3 complex on lignocellulase secretion is conserved in cellulolytic ascomycetes.

**Conclusions:**

Using the model cellulolytic fungus *N. crassa*, we explored potential hypersecretion-related mutations in *T. reesei* strain RUT-C30. Through systematic genetic screening of 86 corresponding orthologous KO mutants in *N. crassa*, we identified several genes, particularly those encoding the AP-3 complex that contribute to lignocellulase secretion. These findings will be useful for strain improvement in future lignocellulase and biomass-based chemical production.

**Electronic supplementary material:**

The online version of this article (doi:10.1186/s13068-015-0302-3) contains supplementary material, which is available to authorized users.

## Background

In recent years, the use of large quantities of inexpensive plant biomass as feedstock for biofuel production has become an increasing focus of research. One of the key steps of the integrated biomass conversion process is production of lignocellulases, which are responsible for the degradation of plant cell wall material into fermentable sugars. This step is one of the major determinants of biofuel production costs [1, 2]. In nature, saprophytic fungi have evolved a highly efficient capability to secrete lignocellulases, with this property subsequently exploited in industry for lignocellulase production [3, 4].

Lignocellulases are synthesized and secreted through fungal secretory pathways [5, 6]. Generally, the nascent peptides of lignocellulases must be translocated into the endoplasmic reticulum (ER) lumen for folding and modification before delivery to the Golgi apparatus for further processing and subsequent targeting to their final proper destination (e.g., the extracellular matrix) via small secretory vesicles [7, 8]. To balance intracellular nutritional homeostasis or relieve the deleterious effects of unfolded or misfolded proteins, lignocellulases may undergo turnover via proteasome- or vacuole/lysosome-mediated degradation processes to meet the demand of nitrogen source recycling within fungal cells [9, 10]. For this reason, the dynamic competition between intracellular protein degradation and secretion may determine the final production titer of lignocellulases.

Currently, the most successful industrial hosts for lignocellulase production are primarily those originating from *Trichoderma* species, for example, *T. reesei* RUT-C30 (ATCC 56765). This hypersecretion mutant was obtained in the late 1970s via a three-step procedure [11–14]: (1) UV mutagenesis of wild-type (WT) Qm6a to generate isolate M7; (2) creation of NG14 by further mutagenesis of M7 using *N*-nitroguanidine; and (3) selection of RUT-C30 following another UV round of mutagenesis on the NG14 strain. Although the final secretion titer has been increased 20-fold in RUT-C30 [11, 15], little is known about the mechanism underlying the physiological hypersecretion process, especially that responsible for remodeling of the secretory pathway and associated regulation. With advances in omics technologies, recent comparative genomic studies have revealed that RUT-C30 contains a large chromosomal fragment deletion and hundreds of small mutations compared with its paternal strain QM6a. Several mutations have affected genes involved in lignocellulase regulation, such as two previously well-characterized targets: the carbon catabolite repression (CCR) regulator *cre1* (tre120117), which was found to be truncated in RUT-C30 [16], and the *gls2α* gene encoding the glucosidase II alpha subunit and engaged in protein glycosylation, which had a frame-shift mutation in RUT-C30 [17]. In addition, several mutations potentially affecting extracellular enzyme trafficking and secretion have also been identified; examples include genes encoding a plasma membrane-related protein (tre81136), a cell wall protein (tre124295), an ARP2/3 complex protein (tre2439), and actin-interacting protein 3 (tre35386) [18, 19]. Although recently reported follow-up work has attempted to explain how these mutations affect phenotype (as defined by the transcriptome and cultivation behavior) [20], direct experimental evidence for the actual ability of each of these targets to contribute to the final protein secretion is still lacking at the cellular level. Although the generation of knockout (KO) mutants for these genes might be a direct way to check whether gene functions contribute to hypersecretion, the construction of hundreds of KO mutants in *T. reesei* would be time consuming and difficult to complete. Given that *Neurospora crassa* has a close phylogenetic relationship with *T. reesei* and possesses a nearly complete set of genome-wide gene deletion mutants, thereby making it a powerful tool for use in genetic studies [21], we reasoned that comparative genomic screening of *N. crassa* mutants could be applied as an alternative approach to study functions of mutated genes in *T. reesei* RUTC-30.

In the present work, systematic screening of 86 *N. crassa* KO mutants for mutated RUT-C30 orthologs was used to identify at least 12 genes with negative effects on lignocellulase secretion and 4 genes with positive effects. We further examined two genes that encode subunits of the adaptor protein 3 (AP-3) complex mediating hypersecretion in *N. crassa* and explored the possible conservation of the underlying mechanism in other ascomycetes including *T. reesei*. On the basis of our findings, we proposed a novel strategy to achieve the hypersecretion of lignocellulase in filamentous fungal systems by disrupting the natural balance between protein secretion and nutritionally required protein degradation.

## Results

### Screening of mutants

**Fig. 1:**
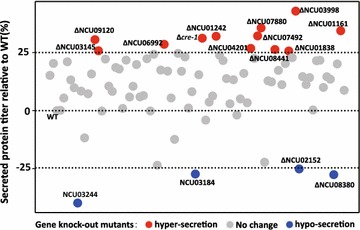
Percentages of secreted protein in 86 *Neurospora crassa* knockout (KO) mutants relative to the wild type (WT). After inoculating conidia from each strain into Avicel medium and culturing for 7 days, the secreted protein titers were measured and are displayed on the *scatter plot*. Mutants with secretion capacities altered by more than 25% compared with the WT are indicated as follows: *red dots* hypersecretion; *blue dots* hyposecretion.

To address whether genes that bear mutations in *T. reesei* RUT-C30 are genuinely involved in protein secretion, we tested the secretion capacity of orthologous gene KO mutants in *N. crassa*. Le Crom et al. [19] previously identified 223 single nucleotide variants, 15 small insertions/deletions, and 18 larger deletions in RUT-C30, with an additional 17 mutations reported by Vitikainen et al. [18]. After excluding mutated genes shown to lack protein secretory functions in the two published studies, 164 mutated *T. reesei* genes were selected for ortholog calling in *N. crassa* using the local BLASTp program. We found 140 orthologs in *N. crassa*, among which 86 had homokaryotic gene KO mutants, including Δ*cre1* (NCU08807) [22]. We further screened these 86 mutants by determining their lignocellulase secretion capacity through batch culturing with microcellulose (2% [w/v] Avicel) as the carbon source and yeast extract (0.75% w/v) as the nitrogen source. Similar to *T. reesei*, lignocellulases accounted for most of the secretome (91% by weight) in *N. crassa*, with the four major components (CBH-1, CBH-2, EG-1, and BG-2) representing about 65% of the total cellulase cocktail proteins [23, 24]. Measured concentrations of extracellular proteins, used to reflect lignocellulase secretion capacity, are shown in Fig. 1 and Table 1. We found lignocellulase production to be reduced by more than 25% in 4 mutants compared with the wild-type strain (WT), with secretions elevated by more than 25% in 12 strains. Similar to Δ*cre1*, 5 of these 12 mutants had markedly increased secretion of lignocellulases, including NCU07880 (annotated as a protein kinase), whose deletion increased the amount of secreted protein by approximately 35% compared with WT *N. crassa* and NCU01242 (encoding a protein predicted as a G2/mitotic-specific cyclin), whose deletion increased protein secretion by about 32%. Both these genes are involved in cell cycle-related functions. In addition, deletion of NCU01161 (encoding a protein functionally similar to actin polymerization protein Bzz1 and associated with endo- or exocytic pathways) increased protein secretion by approximately 34%. Loss of NCU07492, encoding a hypothetical protein, enhanced protein secretion by more than 30%. Finally, loss in *N. crassa* of the NCU03998 gene, whose counterpart in *T. reesei* RUT-C30, tre53811, has a mutation in its exon that changes serine^73^ to leucine [18], increased secreted protein levels up to 42% compared with the WT; this mutant exhibited the highest protein secretion among tested strains. NCU03998 was predicted to encode the μ subunit of the AP-3 complex. Because the way in which the AP-3 complex affects lignocellulase secretion has not been previously reported, we designated the gene at locus NCU03998 as *Ncap3m* in this study and focused on its functional characterization.

**Table 1:** List of extracellular proteins produced by *Neurospora crassa* mutants for genes orthologous to mutation-hit targets in *Trichoderma reesei* RUTC30

No.	Mutant (FGSC)	Orthologs in *N. crassa* ^a^	RUT C30 targets^b^	Gene product names	Protein conc. (mg/L)	Increased vs WT (%)^c^
1	11701	NCU03998	jgi|Trire2|53811|	Adaptor protein complex 3 Mu3A	821.38 ± 21.83	*42.9*
2	17965	NCU07880	jgi|Trire2|124172|	Protein kinase	780.01 ± 32.87	*35.64*
3	19384	NCU01161	jgi|Trire2|68926|	Actin polymerization protein Bzz1	773.06 ± 31.51	*34.44*
4	19393	NCU07492	jgi|Trire2|122689|	Hypothetical protein	758.58 ± 21.01	*32.21*
5	15743	NCU01242	jgi|Trire2|69437|	G2/mitotic-specific cyclin	759.76 ± 41.93	*32.06*
6	10372	NCU08807	jgi|Trire2|120117|	cre-1	755.26 ± 51.79	*31.22*
7	11964	NCU09120	jgi|Trire2|76515|	Lysine-specific histone demethylase Aof2	750.7 ± 20.09	*30.6*
8	15360	NCU06992	jgi|Trire2|31118|	DNA repair protein Nse1	737.31 ± 37.42	*28.59*
9	16681	NCU04201	jgi|Trire2|106234|	Signal transduction protein Syg1	729.66 ± 38.29	*26.84*
10	12039	NCU08441	jgi|Trire2|123786|	Non-ribosomal peptide synthetase	729.27 ± 112.17	*26.36*
11	12276	NCU03145	jgi|Trire2|78301|	Vacuolar membrane zinc transporter	723.79 ± 40.24	*25.81*
12	13177	NCU01838	jgi|Trire2|65106|	Nitrilase	722.38 ± 13.8	*25.71*
13	11468	NCU04142	jgi|Trire2|123114|	Heat shock protein 80	717.96 ± 59.51	24.68
14	12062	NCU07498	jgi|Trire2|79304|	DNA excision repair protein Rad2	715.8 ± 57.68	24.32
15	16319	NCU02842	jgi|Trire2|121087|	Hypothetical protein	712.55 ± 32.87	23.89
16	18830	NCU03811	jgi|Trire2|121453|	Hypothetical protein	710.24 ± 91.52	23.16
17	19831	NCU03545	jgi|Trire2|82153|	Hypothetical protein	706.55 ± 32.91	22.85
18	11956	NCU08809	jgi|Trire2|74765|	Hypothetical protein	704.12 ± 19.44	22.5
19	16594	NCU03914	jgi|Trire2|64375|	Glucan 1,3-beta-glucosidase	704.88 ± 64.43	22.38
20	20296	NCU08364	jgi|Trire2|78320|	Choline sulfatase	702.59 ± 19.06	22.23
21	11040	NCU05411	jgi|Trire2|70071|	Pathway-specific nitrogen regulator	701.46 ± 65.9	21.78
22	19539	NCU07334	jgi|Trire2|68425|	Uracil permease	698.2 ± 25.84	21.72
23	18971	NCU00503	jgi|Trire2|71037|	Nonselective cation channel protein	700.38 ± 66.13	21.59
24	13289	NCU00427	jgi|Trire2|80691|	Hypothetical protein	698.5 ± 19.9	21.52
25	15939	NCU00754	jgi|Trire2|58561|	Multidrug resistant protein	697.71 ± 79.14	21.05
26	13436	NCU05089	jgi|Trire2|64882|	MFS monocarboxylate transporter	692.03 ± 30.99	20.33
27	16570	NCU04886	jgi|Trire2|28409|	MFS multidrug transporter	693.08 ± 69.06	20.3
28	16331	NCU03068	jgi|Trire2|58790|	Glycerol-3-phosphate phosphatase 1	690.98 ± 32.01	20.14
29	20407	NCU11050	jgi|Trire2|75105|	DUF455 domain-containing protein	689.63 ± 40.55	19.86
30	11084	NCU09549	jgi|Trire2|26255|	C6 zinc finger domain-containing protein	683.56 ± 79.47	18.58
31	18230	NCU09887	jgi|Trire2|67030|	Drp1p	678.63 ± 42.93	17.93
32	19296	NCU07276	jgi|Trire2|67732|	ABC bile acid transporter	677.02 ± 69.64	17.5
33	15452	NCU06309	jgi|Trire2|22841|	Hypothetical protein	674.61 ± 18.59	17.36
34	15930	NCU00648	jgi|Trire2|59952|	Choline transporter	675.67 ± 114.38	17.02
35	13757	NCU01420	jgi|Trire2|54157|	Hypothetical protein	672.9 ± 36.3	16.97
36	15627	NCU04626	jgi|Trire2|28731|	G-protein coupled receptor	667.67 ± 37.79	16.05
37	15627	NCU04626	jgi|Trire2|123806|	G-protein coupled receptor	667.67 ± 37.79	16.05
38	16748	NCU00799	jgi|Trire2|40758|	Homocysteine *S*-methyltransferase	669.17 ± 128.93	15.8
39	14141	NCU08499	jgi|Trire2|58161|	GTPase-activating protein GYP5	663.7 ± 13.31	15.49
40	13568	NCU04521	jgi|Trire2|54511|	Hypothetical protein	665.59 ± 127.62	15.19
41	20195	NCU06578	jgi|Trire2|104161|	KapG	662.02 ± 104.79	14.69
42	19233	NCU01997	jgi|Trire2|74570|	ABC transporter	662.83 ± 141.67	14.63
43	12060	NCU07381	jgi|Trire2|3027|	DNA cross-link repair protein pso2/snm1	659.15 ± 93.95	14.25
44	18123	NCU07703	jgi|Trire2|55887|	Hypothetical protein	655.25 ± 49.89	13.82
45	13910	NCU05195	jgi|Trire2|75072|	Hypothetical protein	653.76 ± 52.14	13.55
46	11279	NCU03125	jgi|Trire2|79405|	NIMA-interacting protein TinC	651.64 ± 26.12	13.32
47	12569	NCU00321	jgi|Trire2|67024|	Hypothetical protein	652.72 ± 75.61	13.23
48	19403	NCU07564	jgi|Trire2|78465|	Siderophore iron transporter mirC	648.04 ± 5.97	12.87
49	15566	NCU01961	jgi|Trire2|59147|	DNA lyase Apn2	647.69 ± 69.51	12.39
50	19712	NCU00497	jgi|Trire2|58391|	Hypothetical protein	643.14 ± 129.41	11.27
51	11150	NCU00278	jgi|Trire2|73912|	Hypothetical protein	643.22 ± 144.06	11.2
52	11459	NCU00340	jgi|Trire2|36543|	Transcription factor steA	637.2 ± 73.32	10.54
53	13984	NCU05837	jgi|Trire2|65104|	Vacuolar protein sorting-associated protein 13a	635.14 ± 16.93	10.5
54	19183	NCU05477	jgi|Trire2|102776|	Hypothetical protein	632.43 ± 18.1	10.02
55	11061	NCU08443	jgi|Trire2|77513|	Transcription factor ace3	635.36 ± 137.21	9.87
56	13086	NCU00541	jgi|Trire2|80592|	Hypothetical protein	627.2 ± 73.28	8.8
57	16803	NCU04809	jgi|Trire2|82037|	MFS phospholipid transporter	630.47 ± 248.43	8.4
58	15478	NCU08642	jgi|Trire2|78268|	Cyclic nucleotide-binding domain-containing protein	624.52 ± 83.42	8.28
59	15647	NCU00025	jgi|Trire2|82499|	Integral membrane protein	623.56 ± 73.21	8.17
60	16280	NCU04108	jgi|Trire2|105874|	Isoamyl alcohol oxidase	616.8 ± 25.92	7.26
61	15869	NCU00335	jgi|Trire2|5140|	Pre-mRNA-splicing factor cwc15	612.18 ± 15.31	6.51
62	13160	NCU01633	jgi|Trire2|62380|	Hexose transporter HXT13	611.29 ± 19.85	6.33
63	19733	NCU06832	jgi|Trire2|112231|	Kinesin	609.8 ± 45.91	5.93
64	12341	NCU09864	jgi|Trire2|56726|	2-Oxoisovalerate dehydrogenase alpha subunit	608.18 ± 4.15	5.88
65	11030	NCU07788	jgi|Trire2|52368|	Fungal specific transcription factor	606.86 ± 36.13	5.47
66	12282	NCU06647	jgi|Trire2|5403|	Enoyl-CoA hydratase/isomerase	605.74 ± 7.76	5.43
67	12018	NCU02751	jgi|Trire2|120806|	Serine/threonine-protein kinase	605.22 ± 59.32	5.05
68	11677	NCU01868	jgi|Trire2|59388|	MFS maltose permease MalP	595.74 ± 3.43	3.71
69	18917	NCU05459	jgi|Trire2|65773|	Mitochondrial AAA ATPase	595.8 ± 64.3	3.39
70	19405	NCU07574	jgi|Trire2|22294|	Hypothetical protein	591.48 ± 5.41	2.96
71	17946	NCU04755	jgi|Trire2|45456|	Protein kinase domain-containing protein ppk32	581.14 ± 12.76	1.12
72	17081	NCU01044	jgi|Trire2|63464|	Hypothetical protein	580.08 ± 10.41	0.95
73	19059	NCU08307	jgi|Trire2|56077|	Hypothetical protein	579.21 ± 100.55	0.3
74	17389	NCU02337	jgi|Trire2|80332|	Mitochondrial carrier protein	576.12 ± 32.03	0.14
75	2489	#####	#####	Wild type	575.45 ± 36.81	0
76	16836	NCU04847	jgi|Trire2|52520|	cyclin	573.97 ± 15.22	−0.13
77	20073	NCU06341	jgi|Trire2|44956|	MFS transporter	573.58 ± 9.57	−0.17
78	19245	NCU02220	jgi|Trire2|64866|	hypothetical protein	563.98 ± 19.59	−1.9
79	12078	NCU00523	jgi|Trire2|50268|	NAD-dependent deacetylase sirtuin-2	551.74 ± 23.25	−4.05
80	12072	NCU04203	jgi|Trire2|121351|	Glucosidase II alpha subunit	508.03 ± 35.04	−11.73
81	13475	NCU07119	jgi|Trire2|60458|	Nonribosomal peptide synthase 2	503.65 ± 12.98	−12.37
82	19165	NCU05213	jgi|Trire2|75074|	Hypothetical protein	449.4 ± 86.39	−22.22
83	18185	NCU08452	jgi|Trire2|110570|	Hypothetical protein	439.95 ± 23.92	−23.52
84	16956	NCU02152	jgi|Trire2|3400|	RRM domain-containing protein	430.62 ± 18.69	−*25.11*
85	11357	NCU03184	jgi|Trire2|4921|	C2H2 conidiation transcription factor FlbC	419.08 ± 52.77	−*27.31*
86	20306	NCU08380	jgi|Trire2|122050|	Plasma membrane phosphatase	416.5 ± 18.07	−*27.57*
87	11360	NCU03244	jgi|Trire2|62053|	WD repeat protein	346.47 ± 27.22	−*39.81*

^a^ *N. crassa* orthologs: locus selected according to the *N. crassa* database (v7) (https://www.broadinstitute.org/annotation/genome/neurospora/MultiHome.html).

^b^RUTC30 targets: locus selected according to the *T. reesei* genome sequence web site (http://genome.jgi-psf.org/Trire2/Trire2.home.html).

^c^The increased percentage of secreted protein relative to the WT. Strains more than 25% increased or decreased compared with the WT are shown in italics.

### *Ncap3m* encodes the μ subunit of the AP-3 complex in *N. crassa*

**Fig. 2:**
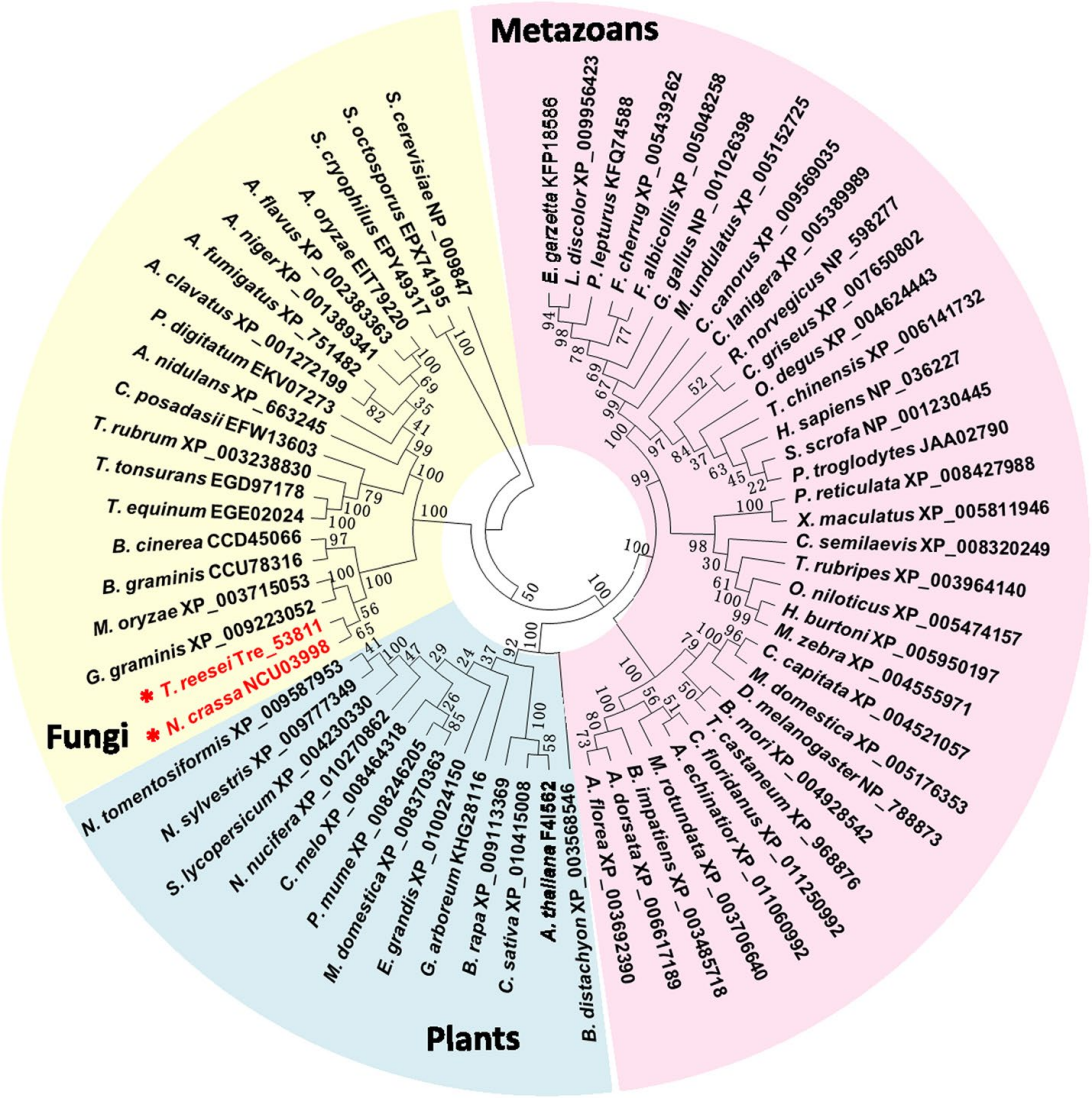
Phylogenetic tree of adaptor protein 3 (AP-3) complex μ-subunit proteins. Amino acid sequences were obtained from the NCBI database based on ortholog calling using the local BLASTp program. The phylogenetic analysis was performed by MEGA6 using the maximum likelihood method with 1,000 bootstrap replicates.

The AP-3 complex is well conserved in various eukaryotes [25, 26]. We were able to identify NcAP3m sequence homologs in *Saccharomyces cerevisiae* (identity: 25%, *E* value: 4 × 10^−15^), *Arabidopsis thaliana* (identity: 32%, *E* value: 2 × 10^−27^), *Drosophila melanogaster* (identity: 34%, *E* value: 9 × 10^−33^), *Mus musculus* (identity: 29%, *E* value: 2 × 10^−32^), and even *Homo sapiens* (identity: 29%, *E* value: 2 × 10^−32^). To reveal the phylogenetic position of *Ncap3m* within eukaryotes, we generated the phylogenetic tree of AP-3 complex μ-subunit proteins shown in Fig. 2. Proteins in this tree were significantly divided into three major groups corresponding to fungi, plants, and metazoans. *Ncap3m* was clustered along with its ortholog tre53811 from *T. reesei* (identity: 57%, *E* value: 0.00) in a well-supported clade within the fungal group.

### *Ncap3m* is involved in lignocellulase production in *N. crassa*

**Fig. 3:**
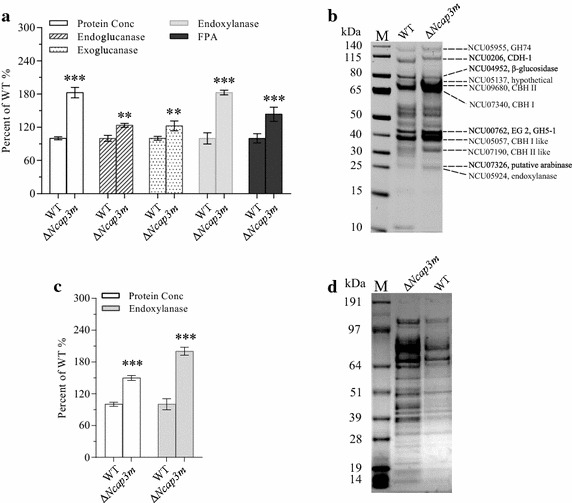
Hypersecretion of lignocellulases by *Neurospora crassa* due to deletion of *Ncap3m*. Typical secretomes of the wild type (WT) and Δ*Ncap3m* are shown on a sodium dodecyl sulfate-polyacrylamide gel electrophoresis (SDS-PAGE) gel (**a**, **c**) after 7 days of culturing in Avicel (**a**, **b**) and xylan (**c**, **d**) media. Total extracellular protein concentration and enzyme activity (**b**, **d**) were measured and evaluated after normalization to the WT control according to percentage (standard error of the mean, *n* = 3). Asterisks indicate significant differences from the WT (**P < 0.01; ***P < 0.001) based on one-way analysis of variance.

To further determine how *Ncap3m* impacts lignocellulase production in *N. crassa*, we performed batch culturing of Δ*Ncap3m* using Avicel or xylan as the sole carbon source, with the WT used as the positive control. In batch cultures with 2% (w/v) Avicel, the protein secretion titer of Δ*Ncap3m* increased by more than 80% compared with the WT. Filter paper activity (FPA) and xylanase activities were, respectively, 44 and 80% higher in the mutant than in the WT, with endoglucanase and exoglucanase activities showing respective increases of approximately 24 and 20% (Fig. 3a). When we changed the culture conditions to 2% xylan as the sole carbon source, thereby allowing xylanase to be specifically induced and secreted [27], the extracellular protein concentration of the Δ*Ncap3m* mutant was improved by approximately 50% and xylanase activity increased by about 100% (Fig. 3c). All of these results were additionally confirmed by sodium dodecyl sulfate-polyacrylamide gel electrophoresis (SDS-PAGE) profiling of the extracellular secretome (Fig. 3b, d), with known cellulase proteins from a previously reported LC–MS analysis used as a reference [23].

**Fig. 4:**
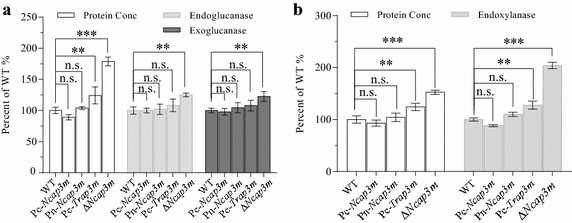
Restoration of the Δ*Ncap3m* hypersecretion phenotype to normal wild-type (WT) levels by *Ncap3m* or *Trap3m*. The following strains were grown in 2% (w/v) Avicel (**a**) or xylan (**b**) media: the WT, the *Ncap3m* gene knockout (KO) mutant (Δ*Ncap3m*), the *Ncap3m*-complemented strain under either the control of the *ccg*-*1* strong promoter (Pc-*Ncap3m*) or the native promoter (Pn-*Ncap3m*), and a *Trap3m*-complemented strain introduced into Δ*Ncap3m* under the control of the *ccg*-*1* strong promoter (Pc-*Trap3m*). After 7 days of culturing, the total extracellular protein concentration and endoglucanase (**a**) or endoxylanase (**b**) activity were measured. Data were normalized to the WT control according to percentage (standard error of the mean, *n* = 3); *asterisks* indicate significant differences from the WT (**P < 0.01; ***P < 0.001; *ns* not significant) based on one-way analysis of variance.

To further show that the lignocellulase hypersecretion phenotype was caused by the loss of *Ncap3m* in *N. crassa* rather than other unknown genetic mutations, additional gene complementation assays were performed. We separately introduced enhanced green fluorescent protein (EGFP)-labeled *Ncap3m* under the control of *ccg*-*1* [28] or native promoters into Δ*Ncap3m*. Batch culturing under either Avicel or xylan utilization conditions revealed that both complemented isolates restored the altered secretion phenotype to levels similar to that of the WT. These results were verified by enzyme activity measurements (Fig. 4).

To investigate whether the hypersecretion observed in *N. crassa* due to the loss of the *ap3m* gene is conserved in *T. reesei*, we conducted an inter-complementation assay by introducing the *ap3m* gene of *T. reesei* into Δ*Ncap3m*. We first cloned the 1,608-bp open reading frame (ORF) of *Trap3m* (tre53811) in the *T. reesei* QM6a cDNA (in-house annotated; see “Methods”; Additional file 1: Figure S1, Additional file 2: Figure S2 for details) and placed it under the control of the *N. crassa ccg*-*1* promoter. The plasmid was then transformed into Δ*Ncap3m:his3*^−^*N. crassa* to form the strain Pc-*Trap3m*. When compared with its parental strain Δ*Ncap3m:his3*^−^, protein secretion and enzyme activities in Pc-*Trap3m* were restored to WT levels, similar to the complemented strain Pc-*ap3m* (Fig. 4). This result implies that *Trap3m* can genetically complement the *ap3m* deletion phenotype in *N. crassa*. Taken together, these results indicate that the function of *ap3m* is evolutionarily conserved between the two lignocellulolytic ascomycetes.

### Sub-cellular localization of the NcAP3m protein in *N. crassa*

**Fig. 5:**
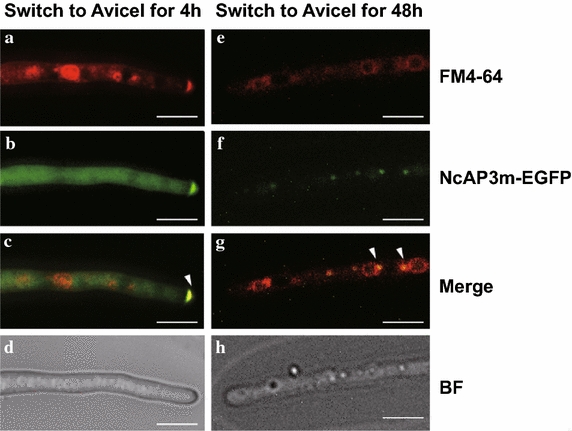
Sub-cellular localization of the adaptor protein 3 (AP-3) complex in *Neurospora crassa.* Wild-type (WT) and Δ*Ncap3m* strains were pre-grown in minimal medium with 2% (w/v) sucrose as the sole carbon source for 16 h and then switched to Avicel medium to elicit lignocellulase production for another 4 h (initial stage; **a**–**d**) or 48 h (logarithmic stage; **e**–**h**). Extreme tip regions of mycelia of the WT and Δ*Ncap3m* were stained with 5 µg/mL of the membrane dye FM4-64 for 30 min to label membrane structures such as the Spitzenkörper or vacuoles (**a**, **e**). Locations of NcAP3m proteins were monitored by recording enhanced green fluorescent protein (EGFP) signal (**b**, **f**). Merged yellow fluorescence signal from FM4-64 and EGFP (**c**, **g**) are denoted by *white arrows* in the photos. Each *scale bar* represents 10 µm.

To assess NcAP3m protein sub-cellular localization, we observed the EGFP signal of NcAP3m–EGFP recombinant protein in young hyphae of the complemented strain Δ*Ncap3m::Ncap3m*–EGFP (Fig. 5). After pre-growth in minimal medium for 16 h followed by culturing in Avicel (2% w/v) medium for another 4 h, we found that NcAP3m–EGFP proteins were unevenly distributed in cytosol; they were primarily located in the extreme tip region of young hyphae, where they accumulated in a ring-like structure. Co-staining with membrane dye FM4-64 showed that NcAP3m–EGFP proteins overlapped with a structure known as the Spitzenkörper that has been reported to serve as a vesicle trafficking center in filamentous fungal cells [29]. This overlap implies that NcAP3m may be located in small secretory vesicles. When the culture time was extended to 48 h, we found that NcAP3m–EGFP proteins no longer accumulated in tip regions. Instead, they were spotted around large vacuole-like structures near the tip region. This observed location is consistent with previous reports showing that the AP-3 complex in higher eukaryotes is primarily located in vacuoles and lysosomes and plays an important role in protein sorting and trafficking between the trans-Golgi network and vacuoles/lysosomes or endosomes [30, 31].

### NCU06569 encoding the β subunit of the AP-3 complex can also affect protein secretion in *N. crassa*

**Fig. 6:**
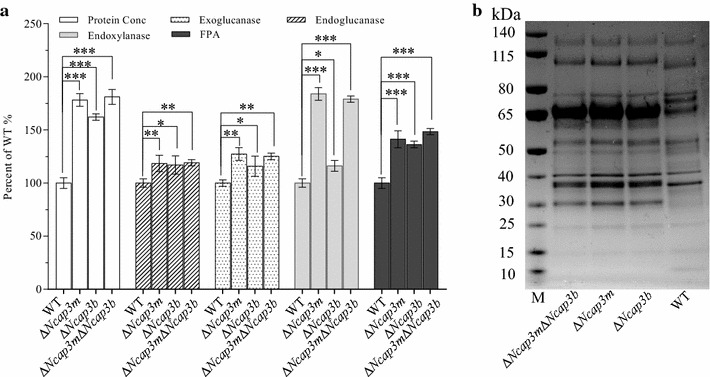
Involvement of the adaptor protein 3 (AP-3) complex in lignocellulase secretion in *Neurospora crassa.* Conidia from the wild type (WT) and *Ncap3m* and *Ncap3b* single (Δ*Ncap3m*, Δ*Ncap3b*) and double mutants (Δ*Ncap3m*Δ*Ncap3b*) were separately inoculated into Avicel medium and batch cultured for 7 days. The typical secretome of each strain was then observed by sodium dodecyl sulfate-polyacrylamide gel electrophoresis (SDS-PAGE) (**b**), while total extracellular protein concentration and enzyme activity (**a**) were measured and displayed after normalization to the WT control according to percentage (standard error of the mean, *n* = 3). *Asterisks* indicate significant differences from the WT (*P < 0.05; **P < 0.01; ***P < 0.001) based on one-way analysis of variance.

Whether other subunits of the AP-3 complex besides NcAP3m can affect protein secretion remain to be explored. Based on genome annotation, three other subunits of the AP-3 complex were found to exist in *N. crassa*; these were separately encoded by NCU06569 (β subunit, *Ncap3b*), NCU04652 (δ subunit, *Ncap3*δ) and NCU09461 (σ subunit, *Ncap3*σ). Among the three genes, the KO mutant was available for *Ncap3b* (FGSC#11856, Δ*Ncap3b*) and we therefore investigated its lignocellulase secretion capacity (Fig. 6a, b). When grown on 2% (w/v) Avicel, the extracellular protein secreted by the Δ*Ncap3b* mutant was about 63% higher than that in the WT; FPA increased by approximately 37%, while endoglucanase, exoglucanase, and xylanase activities increased by about 20% compared with the WT. These results demonstrate that *Ncap3b* also markedly affected protein secretion. We next constructed a double mutant of two subunit-encoding genes of the AP-3 complex (Δ*Ncap3m*Δ*Ncap3b*). This double mutant had an extracellular protein secretion titer and related enzyme activities similar to those of the Δ*Ncap3m* single mutant (Fig. 6a, b), suggesting that NcAP3m is the critical subunit for full functionality of the AP-3 complex. Taking all these results into consideration, we deduced that the AP-3 complex has a significant impact on the secretion of proteins such as lignocellulases.

### Lignocellulase gene transcriptional abundance is maintained at relatively higher levels during the late fermentation phase in AP-3 complex mutants

**Fig. 7:**
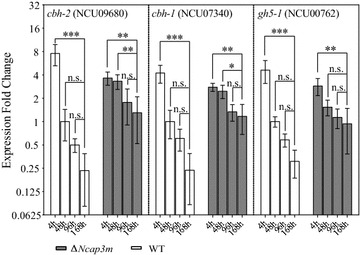
Maintenance of high lignocellulase gene expression levels in Δ*Ncap3m* relative to the wild-type strain (WT) at the late fermentation stage. After Δ*Ncap3m* and WT conidia were grown on Avicel for 4, 48, 96, or 168 h, the transcriptional abundance of three major lignocellulase genes was evaluated by quantitative real-time PCR (qPCR). The data are normalized to the expression of the WT strain at 48 h for each tested gene, with actin (NCU04173) gene expression levels used as an endogenous control in all samples (standard error of the mean, *n* = 3). *Asterisks* indicate significant differences from the control (*P < 0.05; **P < 0.01; ***P < 0.001; *ns* not significant) based on one-way analysis of variance.

To further elucidate the hypersecretion phenotype of the AP-3 complex KO mutants, especially those of the key subunit NcAP3m, we monitored changes in the expressions of lignocellulase genes by quantitative real-time PCR (qPCR) during batch culturing of Δ*Ncap3m*. To avoid potential differences induced by variable growth rates of isolates, shift cultures were used. All strains were pre-grown in 2% (w/v) sucrose until the formation of young hyphae and then switched to Vogel’s medium containing 2% (w/v) Avicel to induce lignocellulase gene expression. We monitored the expression patterns of three genes, namely, *cbh*-*1* (NCU07340), *cbh*-*2* (NCU09680), and *gh5*-*1* (NCU00762), which respectively encode the major exoglucanase, cellobiohydrolase, and endoglucanase proteins in *N. crassa* [23, 24] (Fig. 7). In WT, all three genes had their highest expression values 4 h after induction; expression then rapidly decreased, normally within 48 h, as culturing continued [32]. This phenomenon is known as repressed expression of secreted sequences (RESS), a feedback mechanism that selectively down-regulates transcription of genes encoding extracellular enzymes upon increased protein flux, thereby helping to reduce ER load [33–35]. In Δ*Ncap3m*, however, lignocellulase gene expression levels had not obviously decreased after 48 h and remained at a relatively high level compared with the WT until day 7 (Fig. 7). All of these results indicate that the RESS mechanism might be compromised in Δ*Ncap3m*, even though we still cannot interpret the detailed mechanism at present. This consistently higher induction might be an additional reason for the higher secretion of lignocellulase proteins in AP-3 complex defect mutants.

### Alkaline phosphatase influences lignocellulase secretion

**Fig. 8:**
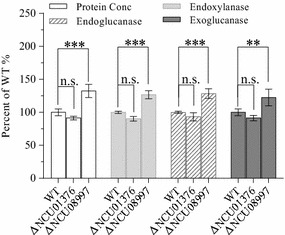
Screenings of two putative alkaline phosphatase knockout (KO) mutants in *Neurospora crassa* to reveal their potential functional association with lignocellulase secretion. Conidia of ΔNCU08997, ΔNCU01376, and wild-type (WT) strains were separately inoculated into Avicel medium and batch cultured for 7 days; total extracellular protein concentration and enzyme activity were then measured and normalized to the WT control according to percentage (standard error of the mean, *n* = 3). *Asterisks* indicate significant differences from the control (**P < 0.01; ***P < 0.001; *ns* not significant) based on one-way analysis of variance.

In *S. cerevisiae*, the AP-3 complex has been shown to be specifically involved in the transport of alkaline phosphatase (ALP) from the Golgi apparatus to vacuoles/lysosomes [30]. Whether ALP affects lignocellulase secretion remains unclear. To address this question, we used the protein sequence of Pho8p, the sole vacuolar ALP in *S. cerevisiae*, as a query in BLASTp searches against the NCBI database (*E* value <1 × 10^−5^), thereby identifying two homologs in the *N. crassa* genome: NCU08997 (identity: 51%, *E* value: 1 × 10^−135^) and NCU01376 (identity: 26%, *E* value: 2 × 10^−17^). Further phylogenetic analysis revealed that the two ALP proteins were also well conserved in other filamentous fungal genomes, including that of *T. reesei* (Additional file 3: Figure S4). When we monitored the lignocellulase secretion capacity of gene KO mutants for the two targets using batch culturing, we discovered that the deletion of NCU08997 had a markedly positive effect on lignocellulase secretion and the deletion of NCU01376 yielded no obvious phenotypes (Fig. 8). This result suggests that NCU08997 serves as the main functional vacuolar ALP in *N. crassa*. Our findings also indicate that ALP involved in efficient protein degradation may be responsible for the observed induction of lignocellulase hypersecretion caused by the AP-3 complex malfunction.

## Discussion

Because lignocellulases are a pivotal factor in the biorefinery process, improvement of their production titers could effectively reduce the cost of biofuel production. Most currently used industrial hosts, such as *T. reesei* (*Hypocrea jecorina*) and *Aspergillus* spp., can achieve an outstanding output; because most of these strains were generated by random mutagenesis, however, a clear genetic basis underpinning the requirements of effective secretion has not yet been elucidated [11]. Previous work comparing the genomic data of two *T. reesei* hyperproducing mutants, NG14 and RUT-C30, with their reference isolate QM6a, uncovered more than 200 mutagenic including many single-nucleotide substitutions, some small insertions/deletions, and more than 100 kb of larger genomic DNA deletions [19]. Some of these mutagenic events have affected genes engaged in functions such as secretion/vacuolar targeting, mRNA stability, and transcription, and thus might contribute to hypersecretion phenotypes. Meanwhile, independent work using high-resolution array comparative genomic hybridization analysis of *T. reesei* NG14 and RUT-C30 uncovered an additional 17 previously undocumented mutation sites. Importantly, two deletions identified in RUT-C30, of a large 85-kb genomic DNA segment and a transcription factor, were both determined to not be involved in cellulase production [18]; this raises the question of whether other mutagenic events have potential roles in boosting secretion capacity. A large-scale screening of each mutation in the *T. reesei* system seems unreachable; however, *N. crassa* is genetically very similar to *T. reesei* and possesses a nearly complete genomic KO strain set, which provides an alternative opportunity to test the possible contribution of chromosomal mutations to effective secretion. In this work, we systematically screened deletion mutants of 86 *N. crassa* genes that were orthologs of *T. reesei* genes having mutations in RUT-C30 relative to its parental strain QM6a. Further examination of the lignocellulase production capacities of these corresponding KO strains by batch culturing revealed 12 strains, including well-known mutant Δ*cre*-*1* [16], which were able to promote protein secretion by more than 25% in comparison with the WT. Among these strains, we found several targets involved in cell cycle-related functions, such as NCU07880, the homolog of the *Aspergillus nidulans* Cdc2-related kinase gene *npkA* [36]; NCU01242, which encodes G2/mitotic-specific cyclin; and NCU06992, which encodes a homolog of the fission yeast DNA repair protein Nse1 possibly contributing to genome stability [37]. Previous work on *T. reesei* has demonstrated that effective secretion is related to low growth rate [38], implying that interruption of the cell cycle may contribute to enhanced lignocellulase secretion. Moreover, NCU01161 encodes an actin polymerization protein that acts as the homolog of *S. cerevisiae* BZZ1 [39]. In filamentous fungi, actin cytoskeleton organization is commonly linked with endo- or exocytic pathways [40]; thus, hypersecretion induced by a defect in NCU01161 might result from alteration of such pathways. In addition, our screening identified several deletion strains, namely, ΔNCU02152, ΔNCU03184, ΔNCU08380, and ΔNCU03244, with decreased secretion capacities, which suggests that they play positive roles in regulating lignocellulase secretion. All four strains were found to have secretion that was decreased by more than 25% compared with WT *N. crassa*, although the detailed mechanisms remain to be elucidated by further studies. Unlike previous observations, we found that deletion of NCU04203, which encodes the alpha subunit of glucosidase II, decreased lignocellulase production by approximately 10% rather than promoting its secretion [17]. We speculate that improperly glycosylated enzyme protein might be degraded intracellularly rather than being secreted to the extracellular matrix. Despite the identification of dozens of mutations in many interesting genes associated with protein synthesis and secretion, it should be noted that our screenings found that most are likely non-functional mutations—at least, no obvious phenotype could be seen at single-gene disruption levels. These results clearly agree with the idea that the hypersecretion phenotype of *T. reesei* RUT C30 is not caused by any single-gene mutation. Because only single-gene KO stocks were screened in this study, the interactions among these mutant alleles could not be elucidated by the present work. However, *N. crassa* can be easily sexually crossed to generate double-, triple-, and multiple-gene mutants, which would help to further investigations of the mechanism of hypercellulase production in RUT C30 in future.

Notably, we found that deletion of *Ncap3m* (NCU03998) in *N. crassa* achieved the highest extracellular protein yield among all screened mutants, with amounts of secreted proteins comparable to Δ*cre*-*1*. Furthermore, the results of FPA and xylanase activity measurements confirm the observed enhancement of cellulase and hemicellulase production in this strain, suggesting that NCU03998 functions in the regulation of secretion without enzyme specificity. Protein sequence alignment revealed that NCU03998 encodes a protein homologous to the AP-3 complex subunit μ3a in mammals and Amp3p in *S. cerevisiae.* Adaptor protein complexes (AP complexes) mediate the formation of vesicles and participate in intra-organelle membrane trafficking in eukaryotes [25, 41, 42]. To date, five AP complexes (AP-1 to AP-5) have been identified [43], and AP-3 has been extensively studied in mammals, flies, Arabidopsis, and yeast [25, 41, 42, 44]. In mammalian cells, the AP-3 complex is a four-subunit heterotetrameric complex consisting of two large subunits (β3a and δ), a medium subunit (μ3a) and a small subunit (σ3) [45, 46]. In addition to *Ncap3m*, we also tested the secretion capacity of another AP-3 complex large-subunit-β3a mutant (ΔNCU06569, Δ*Ncap3b*) in *N. crassa*; this deletion mutant also displayed an enhanced secretion phenotype similar to that of Δ*Ncap3m*, suggesting that the AP-3 complex is indeed involved in lignocellulase secretion. Moreover, we successfully complemented *Trap3m* in Δ*Ncap3m*, implying that the AP-3 complex, and *Trap3m* in particular, may contribute to the hypersecretion phenotype of the *T. reesei* RUT-C30 strain.

**Fig. 9:**
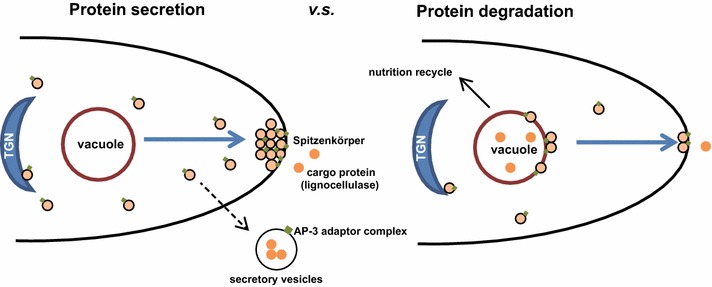
Schematic model illustrating a proposed mechanism for adaptor protein 3 (AP-3) complex-mediated lignocellulase fate decision in *Neurospora crassa*. When *N. crassa* cells are exposed to Avicel at an early stage (4 h), lignocellulases are synthesized and secreted into the extracellular matrix for nutrient acquisition. During this period, the AP-3 complex was observed to accumulate in the tip region—considered the main region for cellulolytic enzyme secretion. As high protein flux tends to temporarily lead to intracellular nitrogen source depletion, partial enzyme molecules at the logarithmic stage of fermentation (48 h) might suffer degradation so that the cell can obtain amino acids through nutrient recycling to maintain normal cell physiological activities such as somatic growth. At this time, the AP-3 complex was found to have re-located to vacuolar membranes, likely mediating delivery of cargo protein (lignocellulases) to secretory vesicles for degradation. Under this scenario, disruption of AP-3 complex function would lead to continued protein secretion, and the hypersecretion phenotype of Δ*Ncap3m* can be reasonably explained.

Previous work on *S. cerevisiae* has demonstrated that the AP-3 complex is required for the intracellular retention of the major chitin synthase Chs3p located in the plasma membrane [47], suggesting that the AP-3 complex can influence secretory protein secretion. Details of the process remain to be elucidated, however, especially in filamentous fungal systems. In addition, the *S. cerevisiae* AP-3 complex has been shown to be specifically involved in the transport of ALP from the Golgi apparatus to vacuoles/lysosomes. Deletion of Amp3p resulted in a pronounced accumulation of ALP in cytoplasm rather than at the vacuolar membrane [30], suggesting that Amp3p plays an important role in ALP location. *PHO8* encodes the sole vacuolar integral membrane ALP in *S. cerevisiae* [48] and is commonly used as a marker protein for monitoring autophagy [49, 50]. ALP can efficiently dephosphorylate a variety of phosphopeptides, thereby releasing phosphate groups [51]. Although the exact physiological function of ALP remains unclear, research using animal models has demonstrated that phosphate can mediate autophagy stimulation [52], which has been partially interpreted to be the potential link between ALP and autophagy. Notably, a previous study of fibroblasts, which are secretory cells that can secrete large quantities of collagen for immunity functions, found that ALP activity can contribute to collagen degradation [53]. That observation raises the possibility that ALP may also contribute to the degradation of other secretory proteins such as lignocellulases in filamentous fungi. In the present study, we found that NcAP3m protein following brief exposure (4 h) of *N. crassa* cells to microcellulose was not located at vacuole/lysosome-like structures as previously reported in eukaryotes. Instead, NcAP3m was located in the tip region of fungal hyphae and likely overlapped with the Spitzenkörper comprising numerous small secretory vesicles. This localization implies that NcAP3m is a critical component of the secretory vesicle membrane and mediates extracellular enzyme secretion. As the culture time was increased to 48 h, rapid lignocellulase synthesis was observed, protein secretion flux increased exponentially, and NcAP3m protein was found to have relocated to large vacuole/lysosome-like structures. As vacuole/lysosome function is usually linked to protein degradation and nitrogen source recycling, we speculate that high rates of secretory protein synthesis and secretion should cause fungal cells to temporarily experience nitrogen source starvation; this in turn would activate the recycling of nutrients via partial lignocellulase degradation. The AP-3 complex consequently seems to balance the secretion and degradation of secretory proteins in fungal cells. Malfunction of the AP-3 complex attenuates the degradation process, which in turn enhances secretion. To check whether ALP contributes to lignocellulase degradation, we also tested lignocellulase secretion capacity in two putative ALP gene KO mutants and found that the deletion of the ALP encoded by NCU08997 led to a hypersecretion phenotype in *N. crassa*. Because the level of hypersecretion was lower than that expected based on the disruption of the AP-3 complex, we hypothesize that other classes of vacuolar enzymes involved in the degradation process may also require the AP-3 complex for correct sorting. Nevertheless, the means by which the AP-3 complex mediates these degradation enzymes locations in *N. crassa* remains unknown and requires further investigation. We found that the deletion of NCU03145, which encodes a vacuolar membrane zinc transporter in *N. crassa*, also enhances lignocellulase secretion. In *S. cerevisiae*, the counterpart of NCU03145 is ZRC1, which functions in transporting zinc from cytosol to vacuole for storage [54]. Vacuolar zinc transporters have been shown to contribute to the maintenance of ALP accumulation and activity [55], implying that the hypersecretion phenotype of ΔNCU03145 might also result from disruption of ALP function. Considering all of these results, we propose a novel model, illustrated in Fig. 9, to explain how the AP-3 complex determines the ultimate fate of lignocellulase.

According to our results, the expression of lignocellulase genes at the transcriptional level had an effect in Δ*Ncap3m* and Δ*Ncap3b*. The typical RESS phenomenon attenuated in these mutants. Although we could not determine the precise mechanism, we speculate that this change may be an indirect consequence of lignocellulase secretion induced by AP-3 complex malfunction, which could yield more efficient degradation of cellulose to produce biopolymer inducers to maintain lignocellulase gene expression. Although the xylanase activity change was proportionate to the protein enhancement level, we additionally noticed that the activity increase of cellulases such as endoglucanase under Avicel conditions, approximately 20%, was not as high as the enhancement of secreted enzymes protein titers (80%). We currently have no good explanation for this observation. As mentioned above, deletion of *S. cerevisiae amp3* resulted in a pronounced accumulation of ALP in the cytoplasm, raising the possibility that ALPs in Δ*Ncap3m* or Δ*Ncap3b* may be secreted in the extracellular matrix, thus modifying lignocellulases and interfering with the enzyme activity.

## Conclusions

In this study, we used gene deletion mutant stocks of *N. crassa* to investigate the function of orthologs of *T. reesei* genes whose mutations might contribute to the hypersecretion phenotype of the RUT-C30 strain. We identified several potential targets, especially the AP-3 complex, involved in the lignocellulase secretion pathway. This study provides a novel view of lignocellulase secretion and suggests a new strategy for future improvement of fungal strains.

## Methods

### Strains

*Neurosporacrassa* strains, including the WT (FGSC#2489) as well as gene KO mutants, were obtained from the Fungal Genetics Stock Center (FGSC; http://www.fgsc.net/) [56]. Double deletion strains (such as Δ*Ncap3m*Δ*Ncap3b*) were generated by performing sexual crosses following previously described protocols [57]. The genotypes of single and double deletion strains were confirmed by PCR as described in Wu et al. [58] using the primers listed in Additional file 4: Table S1. The QM6a strain of *T. reesei* was kindly donated by Dr. Monika Schmoll (Department of Health and Environment-Bioresources, Austrian Institute of Technology).

### Culture conditions and screens for *N. crassa* KO mutants

*Neurospora crassa* stock cultures were maintained on minimal medium agar slants containing 1× Vogel’s salt with 2% (w/v) sucrose [59] at 25°C. For enzyme production, 10-day-old conidia of *N. crassa* strains were suspended in sterile water and inoculated using a final concentration of 1 × 10^6^ per mL into a 250-mL Erlenmeyer flask containing 100 mL medium [60] [1× Vogel’s salt, 2% w/v crystalline cellulose (Avicel PH-101; Sigma-Aldrich, St. Louis, MO, USA), and 0.75% w/v yeast extract (Sigma-Aldrich)]. Flask batch culturing was performed at 25°C under constant light and with agitation (200 rpm) for 7 days; extracellular protein titers were then measured based on the Bradford method. For secreted protein assays, two biological replicates were cultured per strain and three technical replicates were conducted per culture.

### Identification of orthologs and phylogenetic analysis

The protein sequences of genes with potential malfunctions due to various mutations of *T. reesei* RUTC30 were extracted from the latest genome data maintained by the DOE Joint Genome Institute (http://genome.jgi.doe.gov/TrireRUTC30_1/TrireRUTC30_1.home.html) using Perl scripts. Homologs of *N. crassa* proteins were identified using local BLASTp (version Blast+ 2.2.28) with an *E* value <10^−5^ applied as a cutoff. Phylogenetic analysis of *Ncap3m* was carried out in MEGA6 using the maximum likelihood method with 1,000 bootstrap replicates. Database homology searching was performed with the local BLAST program as described above.

### Complementation of Δ*Ncap3m* in *N. crassa*

Complementary plasmids containing the *Ncap3m* coding region under the control of native or strong promoters were constructed separately. For the former case, the 1,566-bp full-length ORF of *Ncap3m* without a stop codon and with a 1,000-bp upstream putative native promoter region was PCR-amplified from WT *N. crassa* genomic DNA using the primers Pnative-F and ap3m-R (Additional file 4: Table S1). After digestion with *Not*I and *Pac*I, the fragment was inserted into plasmid pMF272 [61] and the resulting plasmid was designated as Pn-*ap3m*. For the latter case, the 1,566-bp ORF region of *Ncap3m* was PCR amplified from WT *N. crassa* genomic DNA using the primer set ap3m-F/R (Additional file 4: Table S1), digested with *Xba*I and *Pac*I, and then cloned into the downstream *ccg*-*1* promoter within plasmid pMF272. The resulting plasmid was designated as Pc-*ap3m.* Complementary plasmids were transformed into the host strain with a double deletion (Δ*Ncap3m*:*his3*-) according to the method described in Margolin et al. (http://www.fgsc.net/fgn44/margol.html). Transformants were screened by selection for histidine prototrophy and green fluorescent protein fluorescence of conidia.

### *T. reesei ap3m* gene re-annotation and cloning

For a heterologous functional complementation analysis, we conducted an inter-complementation assay by introducing the orthologs of NCU03998 in *T. reesei* into the *ap3m* mutant. The current ORF of tre53811 (the predicted ortholog of NCU03998) contained 3,658 bp with three introns and encoded a polypeptide of 1,007 amino acids containing three domains: a clathrin adaptor complex small chain, an AP-3 complex medium Mu3, and a malonyl CoA-acyl carrier protein transacylase (Additional file 1: Figure S1). We cloned the homologous gene cDNA of *ap3m* in *T. reesei* QM6a (in-house annotation, modified from jgi_Trire2_53811), consisting of 1,611 bp encoding a polypeptide of 536 amino acids containing the first two domains (amplified with primers Trap3m-F and Trap3m-R) (Additional file 4: Table S1, Additional file 2: Figure S2, Additional file 5: Figure S3). This gene under the control of the *ccg1* promoter was named Pc-*Trap3m* and then transformed into the host strain as described above to yield the selected transformant Pc-*Trap3m*.

### RNA extraction

Ten-day-old conidia of *N. crassa* strains were collected and inoculated into 1× Vogel’s salt solution with 2% sucrose and grown for 16 h. Mycelia were collected, washed several times with 1× Vogel’s salt solution, transferred into medium with 2% Avicel or xylan as the carbon source, and grown to different time points (4, 48, 96, or 168 h). Mycelia were harvested by vacuum filtration, frozen immediately in liquid nitrogen, and stored at −80°C for RNA extraction. Total RNA from frozen samples was isolated using Trizol reagent (Invitrogen Life Technologies, Carlsbad, CA, USA) in accordance with the manufacturer’s protocol. An additional clean-up was performed using an RNeasy mini kit (Qiagen, Hilden, Germany), according to the manufacturer’s RNA Clear Up instructions. RNA integrity and concentration were checked on a Nanodrop instrument and by agarose gel electrophoresis.

### qPCR

Quantitative real-time PCR was performed on a CFX96 real-time PCR detection system (Bio-Rad, Hercules, CA, USA) using reagents supplied with a Toyobo One-Step qPCR kit (Toyobo, Osaka, Japan). The 20-µL reaction mixtures, with three replicates, included 75-ng template RNA, 0.4 µM primers, and 10-µL RNA-direct SYBR Green Real-Time PCR Master Mix. The relative transcript level of each gene was calculated by the $2^{{ - \varDelta \varDelta C_{\text{t}} }}$ method, with its expression in the WT strain used as the control and the expression of the actin gene (NCU04173) used as an internal standard for all experiments. Specific primers for qPCR are listed in Additional file 4: Table S1.

### Protein and enzyme activity measurements

Total extracellular protein content in supernatants was measured using a Bio-Rad DC protein assay kit (Bio-Rad) based on absorbance at 595 nm, with bovine serum albumin used as the standard. FPA was measured by the 3,5-dinitrosalicylic acid method [62]. Exoglucanase activity was measured at 50°C using 1.0 mg/mL *p*-nitrophenyl-β-d-cellobioside (Sigma-Aldrich) in 50 mM citrate buffer (pH 4.8) as the substrate. The reaction mixture containing 250 µL of properly diluted enzyme and 250 µL of 1.0 mg/mL substrate in 50 mM citrate buffer (pH 4.8) was incubated for 10 min at 50°C, and the reaction was terminated by the addition of 500 µL of 1 M Na_2_CO_3_. The release of *p*-nitrophenol (*p*NP) was monitored at an absorbance at 420 nm. The control was the inactivated enzyme, which was boiled at 100°C for 10 min. *p*NP was used to generate a standard curve. In exoglucanase activity analyses, one unit of enzymatic activity was defined as the amount of *p*NP released from the substrate per minute using 1 mL enzyme under the standard assay conditions. Endoglucanase activity in culture supernatants was determined using an azo-cm-cellulose assay kit (Megazyme, Wicklow, Ireland) as described by the manufacturer. Endo-1,4-β-xylanase activities were assayed using an azo-xylan kit (Megazyme) according to the manufacturer’s instructions.

### Fluorescence microscopy and image processing

To localize NcAP3m–EGFP fusion protein using microscopy, the complemented Δ*Ncap3m::Ncap3m*–EGFP strain was inoculated into liquid minimal medium and grown for 16 h. The hyphae were harvested, washed with Vogel’s salt solution, and transferred into inducing medium containing 0.5% (w/v) Avicel for another 4 or 48 h. EGFP fluorescence observations were performed on an Olympus BX51 fluorescence microscopy system. To co-localize the Spitzenkörper, Δ*Ncap3m::Ncap3m*–EGFP cells were also stained with red-fluorescent FM4-64 dye (Invitrogen) at a final concentration of 10 µM in Hanks’ balanced salt solution buffer following the manufacturer’s instructions.

### Statistical analyses

Unless otherwise noted, all experiments were performed in triplicate and statistical tests for significance were determined via one-way analysis of variance using R (version 3.1.1).

## Additional files

Additional file 1: Figure S1. Protein sequence of tre53811 from *Trichoderma reesei* QM6a. Additional file 2: Figure S2. Diagram of tre2_53811 protein from *Trichoderma reesei* QM6a. Additional file 3: Figure S4. Phylogenetic tree of alkaline phosphatase proteins. Additional file 4: Table S1. Primers used in this study. Additional file 5: Figure S3. Protein sequence of the in-house-annotated AP-3 μ subunit from *Trichoderma reesei* QM6a.

## Abbreviations

ALP: alkaline phosphatase
AP-3: adaptor protein complex 3
bp: base pair
CCR: carbon catabolite repression
EGFP: enhanced green fluorescent protein
FM4-64: *N*-(3-triethylammoniumpropyl)-4-(6-(4(diethylamino) phenyl) hexatrienyl) pyridinium dibromide
FPA: filter paper activity
KO: knockout
ORF: open reading frame
*p*NP: *p*-nitrophenol
qPCR: quantitative real-time PCR
RESS: repression under secretion stress
WT: wild type
