# Supplementary material for: Involvement of the adaptor protein 3 complex in lignocellulase secretion in Neurospora crassa revealed by comparative genomic screening
Source: Biotechnol Biofuels. 2015 Aug 20;8:124. doi: 10.1186/s13068-015-0302-3 (PMC4545925; doi:10.1186/s13068-015-0302-3)
Supplement: Additional file 1: Figure S1. — Protein sequence of tre53811 from Trichoderma reesei QM6a. [file 13068_2015_302_MOESM2_ESM.pdf]

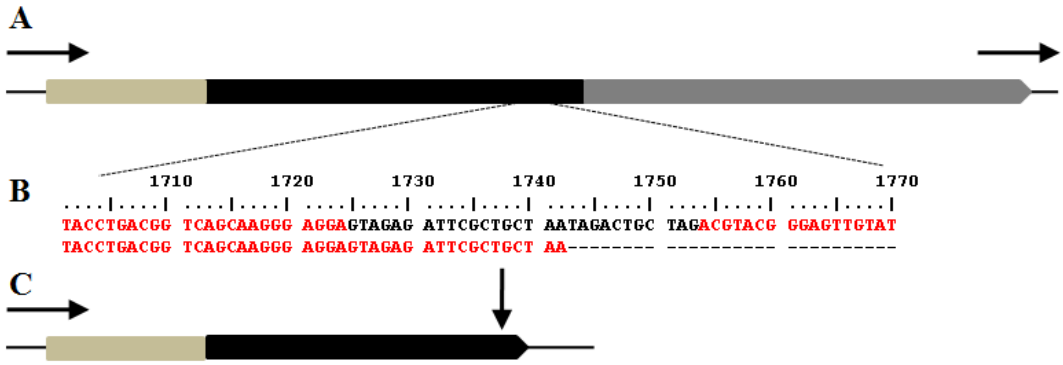

Figure S2 Diagram of tre53811 protein from *Trichoderma ressei* QM6a. A This protein had three domains. First, the clathrin adaptor complex small chain, followed by the adaptor protein (AP) complex AP-3 medium μ3 subunit, and finally malonyl CoA-acyl carrier protein transacylase. **B** Genomic DNA sequence of tre53811 from *T. reesei* QM6a. The predicted gene tre53811 of from *T. reesei* genomic DNA QM6a had five introns; this figure showsthe third intron (black) that we found not to be an intron; instead itcontained a stop codon at 1740 bp. **C** tre53811 protein from *T. reesei* QM6a should have only two domains.
