# Supplementary material for: Involvement of the adaptor protein 3 complex in lignocellulase secretion in Neurospora crassa revealed by comparative genomic screening
Source: Biotechnol Biofuels. 2015 Aug 20;8:124. doi: 10.1186/s13068-015-0302-3 (PMC4545925; doi:10.1186/s13068-015-0302-3)
Supplement: Additional file 2: Figure S2. — Diagram of tre2_53811 protein from Trichoderma reesei QM6a. [file 13068_2015_302_MOESM3_ESM.pdf]

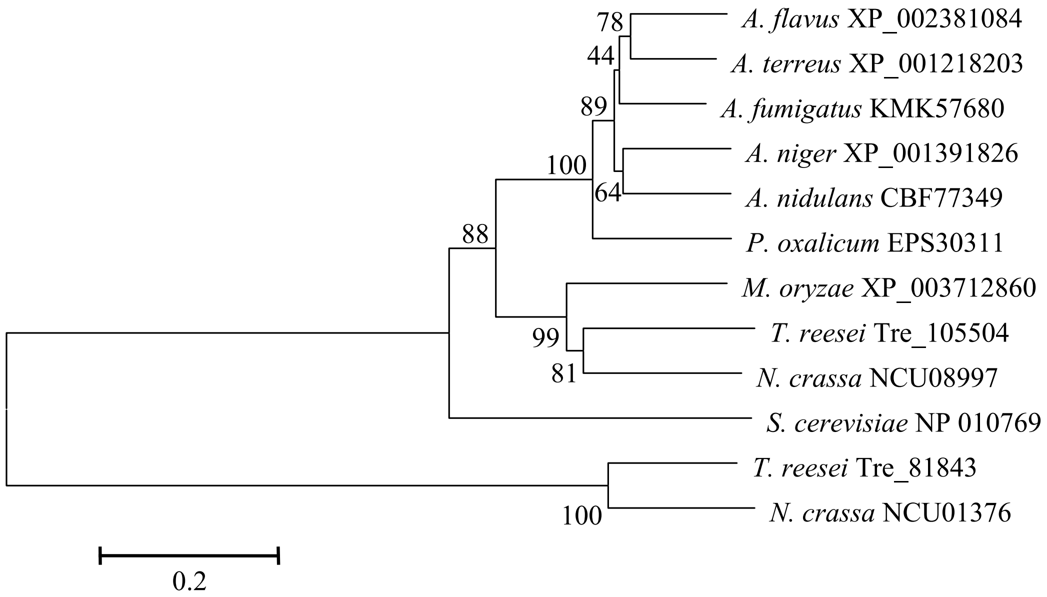

Figure S4 Phylogenetic analysis of the alkaline phosphatase protein. Amino acid sequences were obtained from the NCBI database based on ortholog calling using the localized blastp program. The phylogenetic analysis was done with MEGA 6 using the maximum likelihood method and bootstrap analysis (1,000 replicates) of the ClustalW alignment.
