# Supplementary material for: Involvement of the adaptor protein 3 complex in lignocellulase secretion in Neurospora crassa revealed by comparative genomic screening
Source: Biotechnol Biofuels. 2015 Aug 20;8:124. doi: 10.1186/s13068-015-0302-3 (PMC4545925; doi:10.1186/s13068-015-0302-3)
Supplement: Additional file 3: Figure S4. — Phylogenetic tree of alkaline phosphatase proteins. [file 13068_2015_302_MOESM5_ESM.pdf]

```
1        10        20        30        40        50        60        70
MNGVIEALHIYDDNRNPILSHTYTGRPLSASHLLALYLEHPFPRPSLIYLPNANPPTLVFSLTHSNLLFL
         80        90        100       110       120       130       140
ATSSTEIEPLLVLEFLHRIVDAFEDFVGAPLLAVKLENNYDVIAQLLTEMCDAGTVSTTEPNALREVVEM
         150       160       170       180       190       200       210
EGWVDKLLGSINLPGKSPLNTTPAAPSLIAANTPAVPWRRANVRHTSNELYADVVETLSVTLAPSGRPLA
         220       230       240       250       260       270       280
AFANGTIAFTSKVSGVPDVLVTLGSPSGKHNIGGIMELPVFHPCVRLARWNERPGELSFIPPDGRFILAG
         290       300       310       320       330       340       350
YEVDLLPFTSGKSGSVSSNNLKLPVNLEMKTGLGPVGSEFEVRLQTNKIFGTPNSSAVSQLSRAGVPGRL
         360       370       380       390       400       410       420
SSPHPGSPSSPLLDDLIVTIPLPEDVRNLSDIRPSRGDASFNRAEGRLEWHIPAKEISGPTSHFGLRCTV
         430       440       450       460       470       480       490
VGSLADDEEEEFDPTGFGFGTDYAYNEPYQSTAVKSGKDKAGADDEQDPKKTAQNKILMPSSAAVSFSVK
         500       510       520       530
GWLASGLKIESIVLDSRKSRGLSEGVKPYKGVKYLTVSKGGVEIRC*
```

Figure S3 Protein sequence of in-house annotation AP-3 μ subunit from *Trichoderma reesei* QM6a. This protein had only two domains. One was the clathrin adaptor complex small chain, marked in red, followed by adaptor protein (AP) complex AP-3 medium μ3 subunit, marked in green. This protein contained 536 amino acids.
