# Supplementary material for: Involvement of the adaptor protein 3 complex in lignocellulase secretion in Neurospora crassa revealed by comparative genomic screening
Source: Biotechnol Biofuels. 2015 Aug 20;8:124. doi: 10.1186/s13068-015-0302-3 (PMC4545925; doi:10.1186/s13068-015-0302-3)
Supplement: Additional file 4: Table S1. — Primers used in this study. [file 13068_2015_302_MOESM1_ESM.pdf]

```
1        10        20        30        40        50        60        70
MNGVIEALHIYDDNRNPILSHTYTGRPLSASHLLALYLEHPFPRPSLIYLPNANPPTLVFSLTHSNLLFL
         80        90        100       110       120       130       140
ATSSTETEPLLVLEFLHRIVDAFEDFVGAPLLAVKLENNYDVIAQLLTEMCDAGTVSTTEPNALREVVEM
         150       160       170       180       190       200       210
EGWVDKLLGSINLPGKSPLNTTPAAPSLIAANTPAVPWRRANVRHTSNELYADVVETLSVTLAPSGRPLA
         220       230       240       250       260       270       280
AFANGTIAFTSKVSGVPDVLVTLGSPSGKHNIGGIMELPVFHPCVRLARWNERPGELSFIPPDGRFILAG
         290       300       310       320       330       340       350
YEVDLLPFTSGKSGSVSSNNLKLPVNLEMKTGLGPVGSEFEVRLQTNKIFGTPNSSAVSQLSRAGVPGRL
         360       370       380       390       400       410       420
SSPHPGSPSSPLLDDLIVTIPLPEDVRNLSDIRPSRGDASFNRAEGRLEWHIPAKEISGPTSHFGLRCTV
         430       440       450       460       470       480       490
VGSLADDEEEEFDPTGFGFGTDYAYNEPYQSTAVKSGKDKAGADDEQDPKKTAQNKILMPSSAAVSFSVK
         500       510       520       530       540       550       560
GWLASGLKIESIVLDSRKSRGLSEGVKPYKGVKYLTVSKGGTYGSCIAVLTVDSFRRLEPMIPSNPMPEP
         570       580       590       600       610       620       630
SPRQLRGYPPPATVLIVTKGDPLGSTVLSAPTMLFAASVSNASELTRALRCCAPASGASSAATCPVRWSS
         640       650       660       670       680       690       700
SRPGRPRTAIFFPGQGVQKVGMLSPWLEAFPATASHIIQEIDDVAGFKISDVIQNGPSKVLTQTTMAQPA
         710       720       730       740       750       760       770
IMATSIFILRILEREFDFRVADHFDVTLGHSLGEFTALVAGGYLAFEDSYYLVQRRAAAMSEATKKAIQE
         780       790       800       810       820       830       840
YGGEYGMVAVITEPEYMQPLIKAIRDFVGHSSDGSKSESSEDVPPIEQVLIANINSKNQIVLSGNMERIK
         850       860       870       880       890       900       910
TLIAHVRQFLGHDPRAVRLHSDSPFHSPIMKPAVTVMKNILAKKSRVPGREDEDMVTFPGLMPCISNVSA
         920       930       940       950       960       970       980
RPFESKEQLKDLLARGCLETVRWWASIKYLDQEEKVRRWVGIGPGKVGRNLVGKEVGMRGKDLVKGGGWW
         990       1000
AITDPFEVEEVLRGLEETANIVDDEEE*
```

Figure S1 Protein sequence of tre53811 from *Trichoderma ressei* QM6a. This protein had three domains. First, the clathrin adaptor complex small chain, marked in red, followed by the adaptor protein (AP) complex AP-3 medium μ3 subunit, marked in green, and lastly the malonyl CoA-acyl carrier protein transacylase, marked in blue. This protein contained 1007 amino acids.
