# Supplementary material for: Involvement of the adaptor protein 3 complex in lignocellulase secretion in Neurospora crassa revealed by comparative genomic screening
Source: Biotechnol Biofuels. 2015 Aug 20;8:124. doi: 10.1186/s13068-015-0302-3 (PMC4545925; doi:10.1186/s13068-015-0302-3)
Supplement: Additional file 5: Figure S3. — Protein sequence of the in-house-annotated AP-3 μ subunit from Trichoderma reesei QM6a. [file 13068_2015_302_MOESM4_ESM.pdf]

Table S1 Primer used in this study

Primers	Sequences (5′–3′)	Purpose
Actin-F	CGAGCAGGAGATTCAGACC	qPCR
Actin-R	GCCAGATTCGAGACCAAG	qPCR
NCU08189-F	CAAGATTGATGGCGTGGGACTC	qPCR
NCU08189-R	CGGCATCTCCAACCTCACATC	qPCR
NCU01900-F	CGTGACAAGCAGGGCATCTTC	qPCR
NCU01900-R	CGCCTCGCCATCATCATCAAC	qPCR
NCU02343-F	GGGAAGCAGCGTGTTCAAGG	qPCR
NCU02343-R	CCGTCAGGACCGTCAGTGTAG	qPCR
NCU07225-F	AACGAGGCTGGCGGTCAG	qPCR
NCU07225-R	AGACGGCGAGGTACGAGTTG	qPCR
NCU05924-F	AATGTAAAGGAGGTTGCGGTCAC	qPCR
NCU05924-R	CACGAGTCCGAATCACGAACAC	qPCR
NCU09652-F	GGCGACATCGTGGAGATAGAG	qPCR
NCU09652-R	CAGCCAATCGTCCTTCCAATAAC	qPCR
NCU00762-F	CCAGTACCTCGACTCCGATAGC	qPCR
NCU00762-R	TGGCACCACCAGCGAACTC	qPCR
NCU07340-F	CTATGGCGGTACTTGCGATGC	qPCR
NCU07340-R	CAGCGGAGTCCTTGATGAACTG	qPCR
NCU09680-F	CCAACGGCGAGTTCTCCATC	qPCR
NCU09680-R	GACGAGGTTAGCAAGCGAGTC	qPCR
NCU04952-F	CGTAACTGGGAGGGCTTTGGTC	qPCR
NCU04952-R	GCGGTTTGTCTCCTGTTCGTTG	qPCR
ap3m-F	TCTAGAATGAACGGTGTCATCGAGGC	Gene cloning
ap3m-R	TTAATTAAGCACCTAATCTCCACGCCC	Gene cloning
Pnative-F	GCGGCCGCAGCAGTCTTCGCTAAGGAACATC	Gene cloning
Trap3m-F	CTAGTCTAGAATGAACGGCGTTATTGAAG	Gene cloning
Trap3m-R	CCTTAATTAAGCAGCGAATCTCTACTCCTCCCT	Gene cloning

Underlined regions represent the restriction enzyme sites.
